# Structure-based design and characterization of novel fusion-inhibitory lipopeptides against SARS-CoV-2 and emerging variants

**DOI:** 10.1080/22221751.2021.1937329

**Published:** 2021-06-18

**Authors:** Danwei Yu, Yuanmei Zhu, Tao Jiao, Tong Wu, Xia Xiao, Bo Qin, Huihui Chong, Xiaobo Lei, Lili Ren, Sheng Cui, Jianwei Wang, Yuxian He

**Affiliations:** NHC Key Laboratory of Systems Biology of Pathogens, Institute of Pathogen Biology and Center for AIDS Research, Chinese Academy of Medical Sciences and Peking Union Medical College, Beijing, People’s Republic of China

**Keywords:** SARS-CoV-2, spike protein, membrane fusion, fusion inhibitor, lipopeptide

## Abstract

The ongoing pandemic of COVID-19, caused by SARS-CoV-2, has severely impacted the global public health and socio-economic stability, calling for effective vaccines and therapeutics. In this study, we continued our efforts to develop more efficient SARS-CoV-2 fusion inhibitors and achieved significant findings. First, we found that the membrane-proximal external region (MPER) sequence of SARS-CoV-2 spike fusion protein plays a critical role in viral infectivity and can serve as an ideal template for design of fusion-inhibitory peptides. Second, a panel of novel lipopeptides was generated with greatly improved activity in inhibiting SARS-CoV-2 fusion and infection. Third, we showed that the new inhibitors maintained the potent inhibitory activity against emerging SARS-CoV-2 variants, including those with the major mutations of the B.1.1.7 and B.1.351 strains circulating in the United Kingdom and South Africa, respectively. Fourth, the new inhibitors also cross-inhibited other human CoVs, including SARS-CoV, MERS-CoV, HCoV-229E, and HCoV-NL63. Fifth, the structural properties of the new inhibitors were characterized by circular dichroism (CD) spectroscopy and crystallographic approach, which revealed the mechanisms underlying the high binding and inhibition. Combined, our studies provide important information for understanding the mechanism of SARS-CoV-2 fusion and a framework for the development of peptide therapeutics for the treatment of SARS-CoV-2 and other CoVs.

## Introduction

Coronaviruses (CoVs) are a large group of enveloped viruses with a single positive-stranded RNA genome [1]. Most members of CoVs, including four annually circulating human CoVs (HCov-229E, HCoV-OC43, HCoV-NL63, and CoV-HKU1), infect the respiratory tract of mammals to cause mild disease; however, zoonotic CoVs can cross species barrier from animal reservoirs leading to epidemics in humans with high morbidity and mortality. In 2002, the outbreak of severe acute respiratory syndrome CoV (SARS-CoV) was associated with 8,096 cases and 774 deaths. In 2012, Middle East respiratory syndrome CoV (MERS-CoV) emerged and has so far resulted in >2,500 cases with a ∼35% death rate in 27 countries. The current pandemic of CoV disease 2019 (COVID-19) was caused by SARS-CoV-2, which is a novel CoV genetically close to SARS-CoV [2–4]. While COVID-19 vaccines have been developed and approved for emergency use, it is extremely important to develop effective drugs for combating SARS-CoV-2.

**Figure 1.**
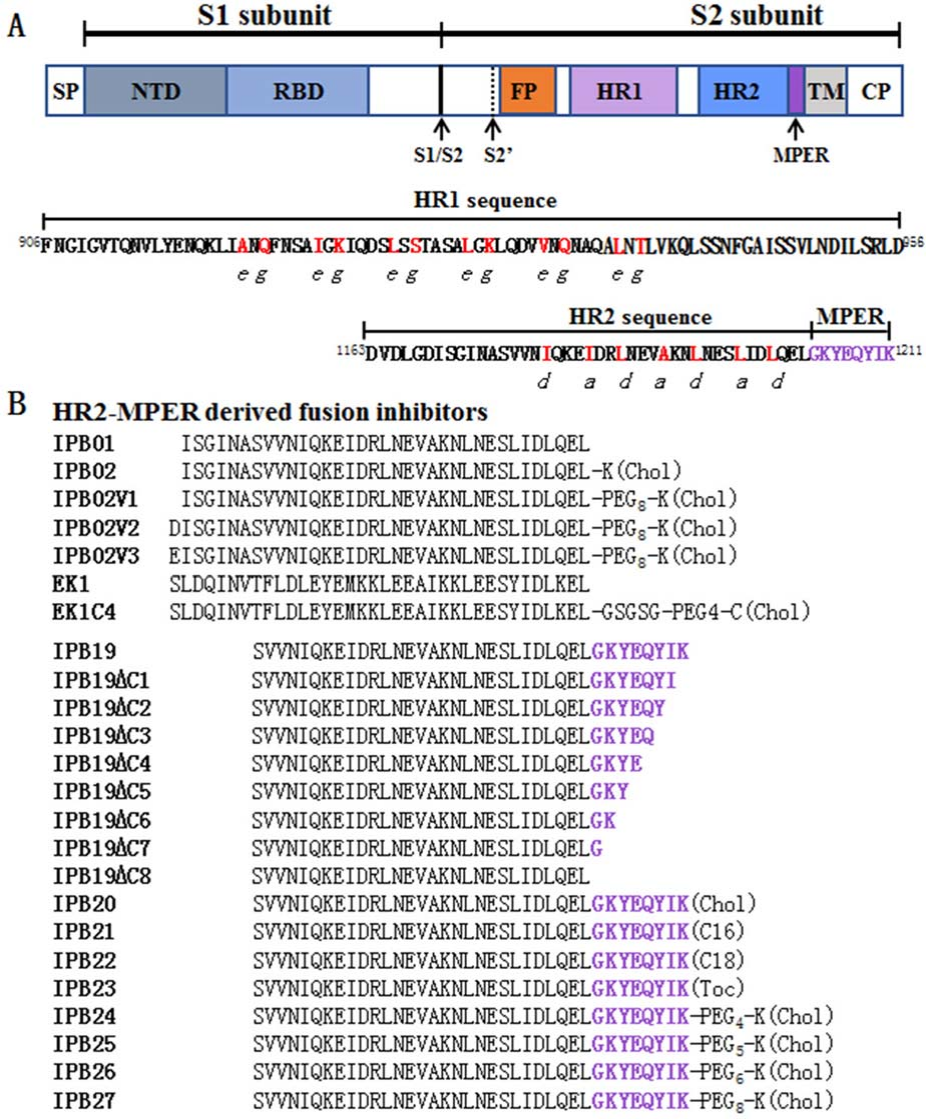
Schematic diagram of SARS-CoV-2 S protein and lipopeptide-based fusion inhibitors. (**A**) Functional domains of the S protein. SP, signal peptide; NTD, N-terminal domain; RBD, receptor-binidng domain; FP, fusion peptide; HR1, heptad repeat 1 region; HR2, heptad repeat 2 region; MPER, membrane-proximal external region; TM, transmembrane domain; CT, cytoplasmic tail. The S1/S2 and S2′ cleavage sites are marked with arrow. The HR1 and HR2 core sequences are listed, in which the potential residues mediating the HR1-HR2 interactions in a 6-HB structure are coloured in red. (**B**) SARS-CoV-2 HR2-MPER derived fusion inhibitor peptides and lipopeptides. The MPER amino acids are coloured in purple. Chol, cholesterol; C16, palmitic acid; C18, stearic acid; Toc, tocophenol; PEG, polyethylene glycol; GSGSG, a flexible amino acid linker.

Like other CoVs, SARS-CoV-2 infection requires membrane fusion between viral envelope and cell membrane, which is mediated by viral trimeric spike (S) glycoprotein [3,5–9]. As illustrated in Figure 1(A), each S protein is subdivided into a receptor-binding S1 subunit and a fusogenic S2 subunit that are delimited by a furin cleavage site (S1/S2), and the S2 contains a second protease cleavage site (S2′) at N-terminus, followed by fusion peptide (FP), heptad repeat-1 domain (HR1), heptad repeat-2 domain (HR2), membrane-proximal external region (MPER), transmembrane domain (TM), and a cytoplasmic tail (CT). During the fusion process, the S protein undergoes dramatic conformational changes: while S1 opens its internal receptor-binding domain (RBD) to interact with the cell receptor angiotensin-converting enzyme 2 (ACE2), the S2 subunit first inserts its FP into the target cell membrane and then assembles HR1 and HR2 into a six-helix bundle (6-HB) structure, which drives the viral and cellular membranes in close apposition for fusion leading to virus entry [5,6].

Peptides derived from HR2 sequences of viral fusion proteins, including the S protein of emerging CoVs, can competitively bind to the HR1 domain and block the formation of the viral 6-HB core, thereby inhibiting infection of the virus from which they were derived [10–12]. However, the previously reported HR2 peptides of CoVs often display low antiviral activities with 50% inhibitory concentrations (IC_50_) in micromolar ranges [13–17]. Emerging studies demonstrate that lipid conjugation to HR2 peptides is a viable strategy to improve the antiviral potency and *in vivo* stability [18–22]. The resulted lipopeptides can interact preferentially with the viral and cellular membranes thus inhibiting virus-cell fusion with elevated local concentrations. While a native HR2 peptide can block direct entry from the cell surface but not entry via the endosomal pathway, lipopeptides also enables activity against viruses that do not fuse until they have been taken up via endocytosis [17,23]. We previously developed a group of HIV fusion-inhibitory lipopeptides with ultrapotent activity [22,24–30], and one (Lipovertide) has been advanced to clinical trials (NCT04592315). In an immediate response to the COVID-19 outbreak, we developed a highly potent SARS-CoV-2 fusion-inhibitory lipopeptide, termed IPB02 [31]. Very recently, we found that IPB02 and its derivatives also exhibited very robust and broad-spectrum inhibitory activity against divergent human CoVs, including SARS-CoV, MERS-CoV, HCoV-229E, and HCoV-NL63 [32].

In this study, we continued our efforts to develop more efficient fusion inhibitor candidates for clinical development. First, the structural and functional properties of the SARS-CoV-2 S2 MPER sequence, a cluster of eight amino acids (^1204^GKYEQYIK^1211^) connecting the HR2 C-terminal (Figure 1), were characterized in terms of its roles in viral infectivity and fusion inhibitor design. Then, a panel of lipopeptides containing the MPER sequence was rationally designed and characterized for antiviral function. We demonstrated that the new inhibitors possess significantly improved activities in inhibiting SARS-CoV-2 and emerging variants, including the B.1.1.7 strain from the United Kingdom and the B.1.351 strain from the South Africa. As anticipated, the new inhibitors also inhibited divergent human CoVs efficiently, confirming that SARS-CoV-2 derived lipopeptides are pan-coronavirus fusion inhibitors. Furthermore, the structural property and binding mechanism of the new inhibitors were analysed by circular dichroism (CD) spectroscopy and crystalographic approach. Taken together, our studies provide important information for understanding the mechanism of SARS-CoV-2 cell fusion/entry and a new strategy for the development of potent and broad-spectrum therapeutics against human CoVs.

## Materials and methods

### Synthesis of peptides and lipopeptides

Peptides were synthesized on rink amide 4-methylbenzhydrylamine (MBHA) resin using a standard solid-phase 9-flurorenylmethoxycarbonyl (FMOC) protocol as described previously [24]. Cholesterol-conjugated lipopeptides were prepared by amidation of a C-terminal lysine side chain with cholesteryl succinate monoester. For fatty acid (C16 or C18) and tocopherol conjugation, the template peptide contained a C-terminal lysine residue with a 1-(4,4-dimethyl-2,6-dioxocyclohexylidene)ethyl (Dde) side chain-protecting group, enabling the conjugation of a C16, C18 or tocopherol that requires a deprotection step in a solution of 2% hydrazine hydrate-*N,N*-dimethylformamide (DMF). All peptides were acetylated at the N-terminus prior to resin cleavage, followed by purification by reverse-phase high-performance liquid chromatography (HPLC) to more than 95% homogeneity and characterized with mass spectrometry.

### Cell-cell fusion assay

A dual-split-protein (DSP)-based fusion cell–cell assay was used to measure SARS-CoV-2 S protein-mediated cell–cell fusion activity and the inhibitory activity of peptides, as described previously [31]. In brief, a total of 1.5 × 10^4^ effector cells (HEK293T) were seeded in a 96-well plate and 1.5 × 10^5^/ml target cells (293T/ACE2) were seeded in a 10-cm culture dish, and then the cells were incubated at 37°C. On the next day, the effector cells were cotransfected with a plasmid expressing the SARS-CoV-2 S protein and a DSP_1-7_ plasmid, the target cells were transfected with a DSP_8-11_ plasmid, and then the cells were incubated at 37°C. After 24 h, a serially 3-fold diluted peptide was added to the effector cells and the cells were incubated for 1 h; the target cells were resuspended at 3 × 10^5^/ml in prewarmed culture medium (Dulbecco's Modified Eagle Medium plus 10% FBS) containing EnduRen live cell substrate (Promega, WI, USA) at a final concentration of 17 ng/ml and incubated for 30 min. Then, 3 × 10^4^ of target cells were transferred to the effector cells and the mixture of cells were spun down to facilitate cell–cell contact. Luciferase activity was measured at different time points using luciferase assay reagents and a luminescence counter (Promega). The data were nonlinearly fitted by GraphPad Prism 6.0 software (GraphPad Software Inc., CA, USA) and 50% inhibitory concentration (IC_50_) was calculated as the final cell culture concentration of an inhibitor that caused a 50% reduction in relative luminescence units (RLU) compared to the level of the virus control subtracted from that of the cell control.

### Single-cycle infection assay

The infectivity and inhibition of pseudoviruses (SARS-CoV-2, SARS-CoV, MERS-CoV, HCoV-NL63, and HCoV-229E) in HEK293T cells that overexpress human ACE2 (293T/ACE2) or Huh-7 cells were determined by a single-cycle infection assay as described previously [31]. To generate pseudoviruses, HEK293T cells were cotransfected with a backbone plasmid (pNL4-3.luc.RE) that encodes an Env-defective, luciferase reporter-expressing HIV-1 genome and an S protein-expressing plasmid. After 48 h, cell culture supernatants containing virus particles were harvested, filtrated, and stored at −80°C. To detect the inhibitory activity of peptides, pseudoviruses were mixed with an equal volume of a serially 3-fold diluted peptide and incubated at 37°C for 30 min. The mixture was then added to 293T/ACE2 or Huh-7 cells at a density of 10^4^ cells/100 μl per plate well. After incubation at 37°C for 48 h, the cells were harvested and lysed in reporter lysis buffer, and luciferase activity was measured and IC_50_ values were calculated as described above

### Inhibition of live SARS-CoV-2 infection

Vero cells (African green monkey kidney cell) were seeded in 96 wells plate one day before infection at a concentration of 1 × 10^4^ cells/well. Live SARS-CoV-2 at MOI of 0.1 was mixed with a serially 3-fold diluted inhibitors and incubated at 37°C for 1 h. After the cells were washed with opti-MEM for one time, the virus-inhibitor mixture was added and incubated at 37°C for 1 h. Then, the mixture was replaced with opti-MEM containing 1% bovine serum albumin (BSA) and cultured at 37°C for 24 h. Culture supernatants were collected and viral RNA were extracted by using Direct-zol RNA MiniPrep kit (Zymo Research, CA, USA) according to the manufacturer's instructions. Viral copy numbers (VCN) were measured by RT–PCR using primers and probe targeting the SARS-CoV-2 N gene. PCR amplification cycle was 50°C, 15 min, 95°C, 3 min; 95°C, 15 s, 60°C, 45 s+ Plate Read, 50 cycles. The copies of the virus were calculated according to the standard curve. Percent inhibition was obtained by dividing the number of copies of the virus in the vehicle control group, and the IC_50_ of each inhibitor was calculated by GraphPad Prism 6.0 software.

### Site-directed mutagenesis

The SARS-CoV-2 spike mutants was generated by site-directed mutagenesis. Brieﬂy, two primers were designed to contain specific mutation and occupied the same starting and ending positions on the opposite strands of a wild-type (WT) *S*-expressing plasmid. DNA synthesis was conducted by PCR in a 50-μl reaction volume using 100 ng of denatured plasmid template, 50 pM upper and lower primers, and 5 U of the high-fidelity polymerase PrimeStar (TaKaRa, Dalian, China). PCR amplification was done for one cycle of denaturation at 98°C for 5 min, followed by 25 cycles of 98°C for 10 s and 68°C for 9 min, with a final extension at 72°C for 10 min. The amplicons were treated with restriction enzyme DpnI for 3 h at 37°C, and DpnI-resistant molecules were recovered by transforming *Trans 2*-Blue Chemically Competent Cell (TransGen Biotech, Beijing, China) with antibiotic resistance. The required mutation was confirmed by DNA sequencing.

### Cytotoxicity of inhibitors

The cytotoxicity of lipopeptide fusion inhibitors on 293T/ACE2 and Huh-7 cells was measured using a CellTiter 96 AQueous One Solution cell proliferation assay (Promega). In brief, 50-μl volumes of lipopeptides at graded concentrations were added to cells, which were seeded on a 96-well tissue culture plate (1 × 10^4^ cells per well). After incubation at 37°C for 2 days, 20 μl of CellTiter 96 AQueous One solution reagent was added into each well and incubated 2 h at 37°C. The absorbance was measured at 490 nm using a SpectraMax M5 microplate reader (Molecular Devices, San Jose, CA, USA), and cell viability (percentage) was calculated.

### CD spectroscopy

Circular dichroism (CD) spectroscopy was used to detect the secondary structure and thermostability of peptides or peptide complexes as described previously [31]. Briefly, a peptide was dissolved in phosphate-buffered saline (PBS; pH 7.2) with a final concentration of 10 μM and incubated at 37°C for 30 min. CD spectra were obtained on Jasco spectropolarimeter (model J-815) a using a 1 nm bandwidth with a 1 nm step resolution from 195 to 270 nm at room temperature. The spectra were corrected by subtracting a solvent blank, and the α-helical content was calculated from the CD signal by dividing the mean residue ellipticity [*θ*] at 222 nm by with a value of −33,000 deg cm^2^ dmol^−1^, corresponding to a 100% helix. Thermal denaturation was done by monitoring the ellipticity change at 222 nm from 20 to 98°C at a rate of 2°C/min, and the melting temperature (*T_m_*) was defined as the midpoint of the thermal unfolding transition.

### Crystallization and structure determination

The 6-HB containing IPB19 and N52 was prepared by dissolving an equal amount (1:1 molar ration) of the peptides in the denaturing buffer (100 mM NaH_2_PO_4_, 10 mM Tris-HCl, pH 8.0, 8 M urea). The mixture was dialyzed against the buffer containing 50 mM Tris-HCl, pH 8.0, 100 mM NaCl at 4°C overnight to allow refolding. The resulting sample was subjected to the size-exclusion chromatography (Superdex75 10/300 GL, GE Healthcare) to collect the predominant peak corresponding to the size of the 6-HB. The complex peptide was crystallized by mixing equal volume (1 μl) of the purified sample (10mg/ml) and the reservoir solution containing 1.26M Sodium phosphate monobasic monohydrate, 0.14M Potassium phosphate dibasic, pH 5.6 in a hanging drop vapour diffusion system at 23 degree. The cryocooling of the crystals was achieved by soaking the crystals 30–60 s in the reservoir solution containing 15% glycerol followed by flash freezing in liquid nitrogen. Complete datasets were collected on beamline BL19U at the Shanghai Synchrotron Research Facility (SSRF) with x-ray wavelength of 0.98 Å. The crystal belonged to the space group of H32, contained one IPB19 and one N52 peptides compring one third of a complete 6-HB per asymmetry unit, and diffracted the x-ray to the resolution limit of 1.24 Å. The structure was solved by molecular replacement (Phaser for MR, CCP4 package) using the crystal structure of post fusion core of SARS-CoV-2 S2 subunit (Protein Data Bank code 6LXT) as the searching model. The structure was refined using PHENIX. The final atomic model has excellent refinement statistics and stereochemistry qualities and validated by MolProbity analysis (Table S1). The MolProbity score for the structure is 1.28, rating 91th percentile among structures of comparable resolution. The Ramachandran plot finds all residues in the favoured area. All structure figures were generated with the programme Pymol (Schrodinger, NY, USA).

## Results

### Structural and functional characterization of the fusion protein MPER sequence

**Figure 2.**
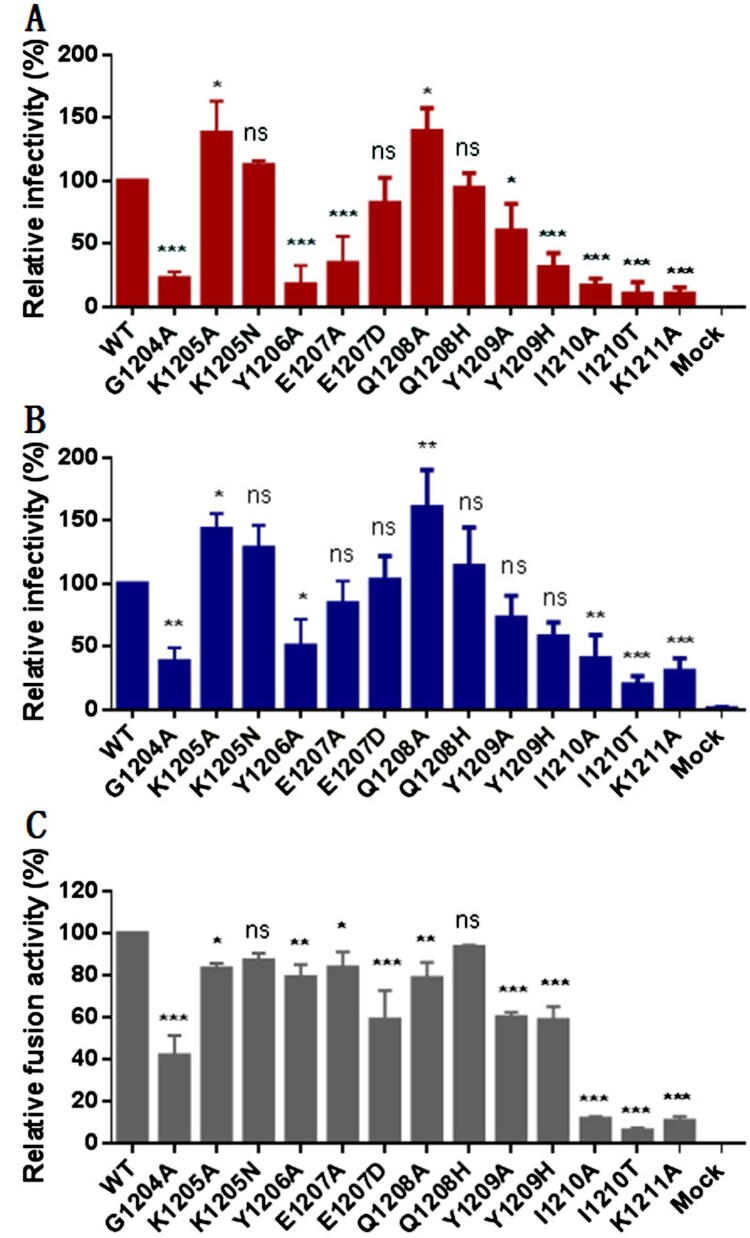
Mutational analysis of the SARS-CoV-2 spike MPER sequence. Infectivity of the SARS-CoV-2 mutant pseudoviruses in 293T/ACE2 cells (**A**) or Huh-7 cells (**B**) was determined by a single-cycle infection assay. (**C**) Fusogenic activity of the SARS-CoV-2 S mutants was determined by a DSP-based cell fusion assay. In comparison, the luciferase activity (RLU) of the wild-type (WT) was treated as 100% and the relative infection or fusion of a mutant was calculated accordingly. The experiments were repeated three times and data are expressed as the means ± standard deviations (SD). Statistical comparison was conducted by *t*-test (*, *P*<0.05; **, *P*<0.01; ***, *P*<0.001; ns, not significant).

Our group and others recently reported several HR2 peptide-based SARS-CoV-2 fusion inhibitors, in which the peptide sequence corresponding to IPB02 (Figure 1) was exclusively used as a design template [31,33,34]. Herein, we wondered whether the MPER sequence in the HR2 C-terminal of the S2 fusion protein could be applied for fusion inhibitor design, thus initiating a project to characterize its structural and functional effects. First, each of the MPER residues was mutated to alanine (alanine scanning) or a naturally-occurring amino acid and impacts of the mutations on the functionality of spike were assessed. As determined by a pseudovirus-based single-cycle infection assay and a dual-split protein (DSP)-based cell–cell fusion assay, a number of mutations resulted in significantly reduced infectivity and cell fusion capacity relative to the wild-type (WT) S protein, while two mutants (K1205A and Q1208A) enhanced the pseudovirus entry but not the cell–cell fusion (Figure 2).

**Figure 3.**
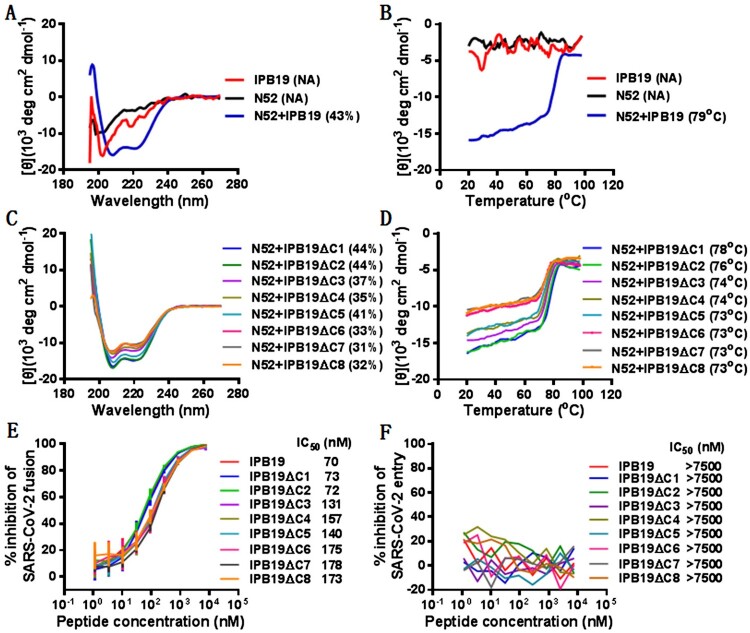
Structural and functional characterization of HR2-MPER derived peptide IPB19. The α-helicity (**A**) and thermostability (**B**) of IPB19 alone or in complex with an HR1-derived target mimic peptide N52 as well as the α-helicity (**C**) and thermostability (**D**) of C-terminally truncated IPB19 peptides complexed with N52 were determined by CD spectroscopy, with the final concentration of each peptide being 10 μM. The inhibitory activities of IPB19 and its truncated versions on S protein-mediated cell-cell fusion (**E**) and pseudovirus infection in 293T/ACE2 cells (**F**) were determined by DSP-based fusion assay and single-cycle infection assay, respectively. The CD experiment was performed two times and obtained consistent results and representative data are shown. The antiviral experiments were repeated three times, and data are expressed as the means ± SD.

We next synthesized an HR2 peptide containing the MPER sequence, termed IPB19, and its structural property in the presence or absence of an HR1-derived target mimic peptide (N52) was characterized by CD specroscopy. As shown in Figure 3(A,B), IPB19 and N52 alone displayed no or minor α-helicity with undefined melting temperature (*T*_m_) values, but they interacted each other to form a typical α-helical complex, which exhibited a helical content of 43% and a *T*_m_ of 79°C. In order to further define the functionality of the MPER motif, a panel of C-terminally truncated peptides (IPB19ΔC1∼IPB19ΔC8) was synthesized and characterized. As shown in Figure 3(C,D), all truncated peptides interacted with N52 to form α-helical complexes, and in comparison, the IPB19ΔC3/N52 complex exhibited a markedly reduced *T*_m_ value (74°C), suggesting that three amino acids (YIK) in the C-terminal of IPB19 critically determined the binding stability.

The inhibitory activities of IPB19 and its truncated peptides on S protein-mediated cell fusion and SARS-CoV-2 pseudovirus were then determined. As shown in Figure 3(E), all of IPB19-based peptides efficiently inhibited the cell–cell fusion with IC_50_ values ranging from 70 to 173 nM, and deletion of the YIK motif attenuated the inhibitory activity. However, the peptides, even at a concentration as high as 7500 nM, did not show significant inhibition on the infection of SARS-CoV-2 pseudovirus in 293T/ACE2 cells (Figure 3(F)), in agreement with our previous findings: the unconjugated peptides such as IPB01 and EK1 could potently block the S protein fusion capacity but not the pseudovirus infection.

### Design and characterization of novel SARS-CoV-2 fusion inhibitor lipopeptides

**Figure 4.**
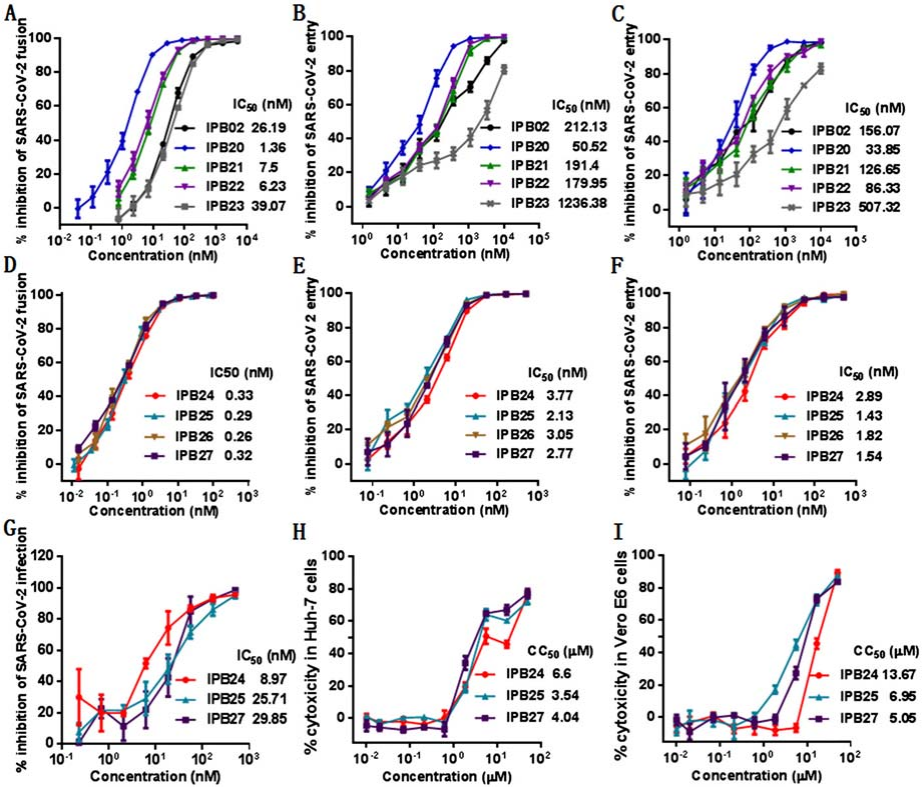
Inhibitory activity of IPB19-based lipopeptides against SARS-CoV-2. The inhibitory activities of IPB19-based lipopeptides on the SARS-CoV-2 S protein-mediated cell-cell fusion (**A** and **C**) and pseudovirus infections in 293T/ACE2 cells (**B** and **E**) or Huh-7 cells (**C** and **F**) were determined by DSP-based cell fusion assay and single-cycle infection assay, respectively. The inhibitory activity of three lipopeptides against live SARS-CoV-2 infection in Vero cells (**G**) as well as their cytoxicity on Huh-7 cells (**H)** or Vero cells (**I**) were respectively determined. The experiments were repeated three times, and data are expressed as the means ± SD.

Given that the YIK motif in MPER contributed to the binding and inhibitory capacities of IPB19, we sought to design IPB19-based fusion inhibitor lipopeptides with improved antiviral activity. First, IPB19 was C-terminally conjugated with different classes of lipids including cholesterol (Chol), palmitic acid (C16), stearic acid (C18), and α-tocopherol (Toc), generating four new lipopeptides (IPB20∼IPB23) as illustrated in Figure 1(B). The inhibitory activity of the IPB19-based lipopeptides along with the previously reported IPB02 on SARS-CoV-2 was examined by the cell–cell fusion and single-cycle infection assays. As anticipated, all inhibitors could potently inhibit S protein-mediated cell fusion and pseudovirus infections in both 293T/ACE2 and Huh-7 cells, with the cholesterol-modified IPB20 showing the highest efficacy (Figure 4(A–C)). Comparing the two inhibitors with direct cholesterol conjugation, IPB20 was about 4∼7-fold more active than IPB02, suggesting that the IPB19 sequence is a more suitable template than the peptide sequence of IPB02 in designing of SARS-CoV-2 fusion inhibitors.

Our previous studies found that introducing a flexible linker between the peptide sequence and cholesterol molecule could markedly improve the antiviral potency of IPB02-based lipopeptides [32]. Thus, we further generated four lipopeptides (IPB24, IPB25, IPB26, and IPB27) by adding different lengths of polyethylene glycol (PEG_4_, PEG_5_, PEG_6_, and PEG_8_) into IPB20 (Figure 1(B)). Promisingly, all new lipopeptides with a linker exhibited dramatically increased activity in inhibiting the S protein cell fusion and SARS-CoV-2 pseudovirus infection, as indicated by their 12∼24-fold decreased IC_50_ values (Figure 4(D–F)). Furthermore, the potency of three potent inhibitors (IPB24, IPB25, and IPB27) was validated with authentic SARS-CoV-2. As shown in Figure 4(G), IPB24, IPB25, and IPB27 inhibited SARS-CoV-2 infection in Vero E6 cells with mean IC_50_ values of 8.87, 25.71, and 29.85 nM, respectively. The 50% cytotoxic concentration (CC_50_) of IPB24, IPB25, and IPB27 was simultaneously measured in Huh-7 and Vero E6 cells, and the results demonstrated their relatively high selectivity indices defined as the ratio of CC_50_ to IC_50_. Taken together, these results suggested that the newly designed lipopeptides containing the MPER sequence are the most potent fusion inhibitors against SARS-CoV-2, in which a relatively short linker is required for achieving their high antiviral activities.

### New lipopeptides are highly potent inhibitors of divergent SARS-CoV-2 variants

**Figure 5.**
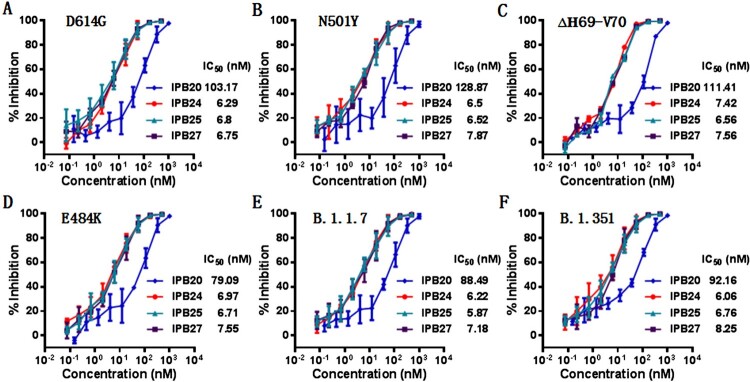
Inhibitory activity of new lipopeptides against emerging SARS-CoV-2 variants. The inhibitory activities of IPB19-based lipopeptides on the SARS-CoV-2 pseudoviruses bearing the S protein with the emerging mutaions of D614G (**A**), N501Y (**B**), Δ69-70 (**C**), E484K (**D**), N501Y/Δ69-70/P681H (**E**), and N501Y/E484K/K417N (**F**) were determined in Huh-7 cells by the single-cycle infection assay. The experiments were repeated three times, and data are expressed as the means ± SD.

The evolutionary changes of SARS-CoV-2 are one of utmost concerns over the ongoing pandemic. The D614G mutation, which locates at the carboxyl (C)-terminal region of the S1 subunit, can render the virus with significantly increased infectivity, and the mutant has already outcompeted the original virus and become a globally dominant circulating form [35,36]. Very recently, the emergence of rapidly-spreading SARS-CoV-2 variants in the United Kingdom (B.1.1.7), South Africa (B.1.351), and elsewhere with mutations in the D614G background has raised tremendous concerns for escape from neutralizing antibody responses and loss of vaccine efficacy [37,38]. The S protein of B.1.1.7 contains 8 mutations: one mutation (N501Y) within the RBD, three mutations (ΔH69-V70, ΔY144,and A570D) within S1, and four mutations (P681H, T716I, S982A, and D1118H) within S2, whereas the S protein of the B.1.351 variant harbours three pivotal mutations (K417N, E484K, and N501Y) within the RBD. While considerable efforts have been devoted to characterizing the effects of the mutations on the efficacies of vaccines and neutralizing antibodies, little is known about the impacts of the emerging variants on the antiviral activity of SARS-CoV-2 fusion inhibitors. To this end, we constructed a panel of pseudoviruses with the S proteins carrying the major mutations of the B.1.1.7 and B.1.351 variants, and their susceptibility on the new inhibitors was determined by the single-cycle infection assay. As shown in Figure 5, the inhibitory activities of IPB20, IPB24, IPB25, and IPB27 were not significantly affected by divergent mutants, including D614G, E484K, N501Y, Δ69–70, B.1.1.7 (N501Y/Δ69–70/P681H), and B.1.351 (N501Y/E484K/K417N).

### New lipopeptides possess broad-spectrum inhibitory activity against other human CoVs

**Figure 6.**
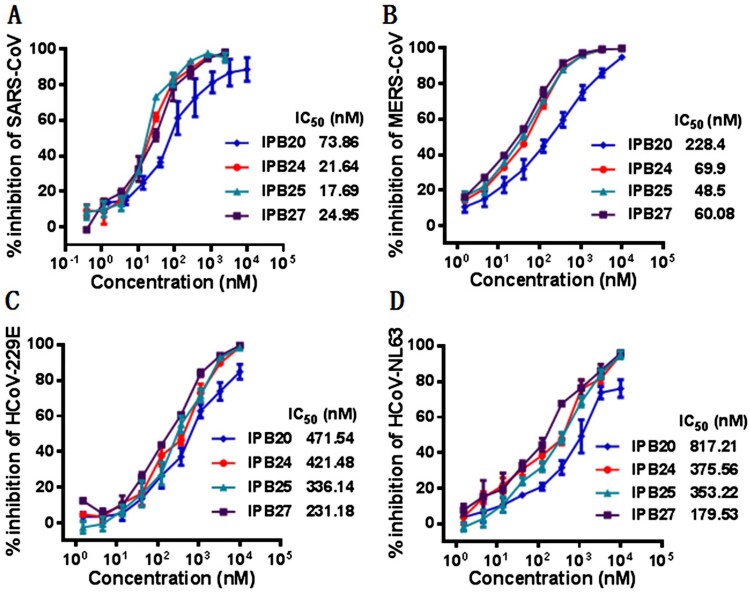
Broad-spectrum inhibitory activity of IPB19-based lipopeptides against divergent human CoVs. The inhibitory activity of IPB19 derivatives against the SARS-CoV (**A**), MERS-CoV (**B**), HCoV-NL63 (**C**) and HCoV-229E (**D**) pseudovirus infections in Huh-7 cells was determined by single-cycle infection assays. The experiments were repeated three times, and data are expressed as the means ± SD.

We previously demonstrated that IPB02-based lipopeptides exhibited potent inhibitory activity against other human CoVs, including SARS-CoV, MERS-CoV, HCoV-229E, and HCoV-NL63. In order to characterize whether the IPB19 derivatives also possess a broad-spectrum anti-CoV activity, the pseudoviruses with the S proteins of SARS-CoV, MERS-CoV, HCoV-229E, and HCoV-NL63 were prepared and single-cycle infection assay was similarly conducted in Huh-7 cells. As anticipated, while IPB20 inhibited the corresponding pseudoviruses of four human CoVs with IC_50_ ranging from 73.86–817.21 nM, three lipopeptides with the linkers (IPB24, IPB25, and IPB27) displayed increased potencies, as indicated by their IC_50_ ranging from 17.69 to 421.48 nM (Figure 6). For example, IPB25 inhibited SARS-CoV and MERS-CoV with IC_50_ of 17.67 and 48.5 nM, respectively. Taken together, these results confirmed that SARS-CoV-2 derived lipopeptides are pan-coronavirus fusion inhibitors.

### Structural characterization of fusion inhibitor lipopeptides

**Figure 7.**
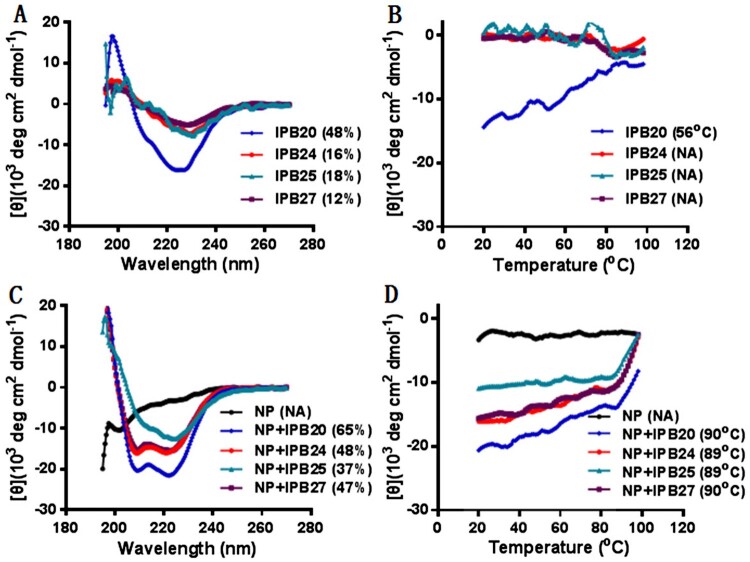
Structural properties of new lipopeptides determined by CD spectroscopy. The α-helicity and thermostability of lipopeptides alone (**A** and **B**) or in complex with HR1 peptide N52 (**C** and **D**) were determined by CD spectroscopy, with the final concentration of each peptide being 10 μM. The experiment was performed two times and obtained consistent results and representative data are shown.

The structural properties of IPB19-based lipopeptides were first characterized by CD spectroscopy. As shown in Figure 7, IPB20 alone displayed α-helicity with a helical content of 48% and a *T*_m_ of 56°C, but incorporation of the PEG linkers sharply reduced the α-helicity of three lipopeptides (IPB24, IPB25, and IPB27). Nonetheless, each inhibitor could interact with the target mimic peptide N52 forming α-helical complexes, which showed very high thermostabilities with *T*_m_ values of ∼90°C.

**Figure 8.**
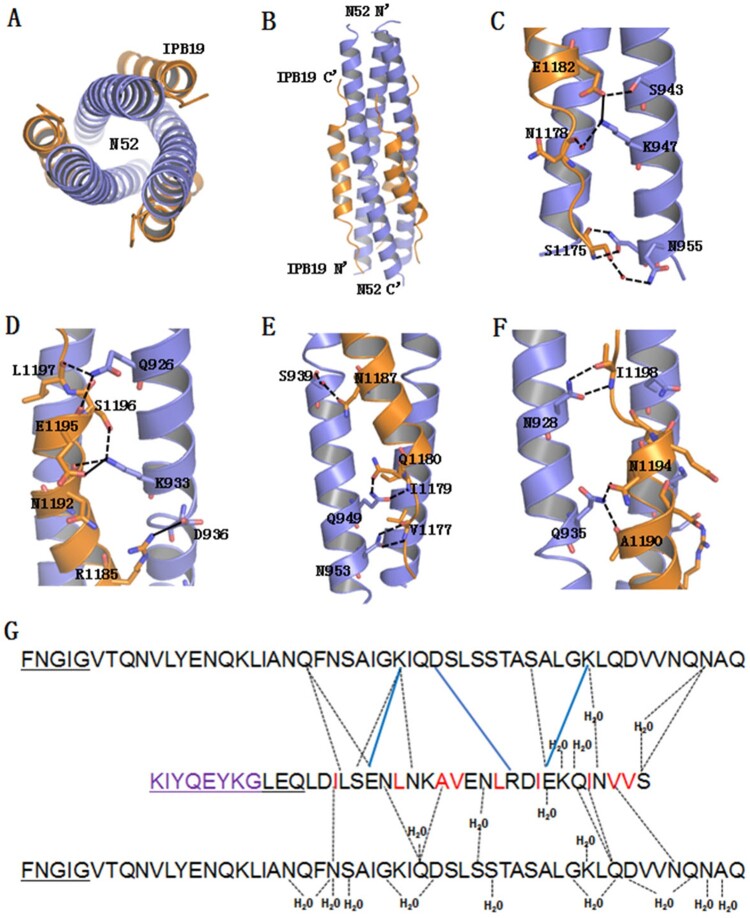
Crystal structure of IPB19 complexed with N52. (**A** and **B**) The overall structure of IPB19/N52-based six-helical bundle (6-HB) is shown in a ribbon model and viewed from the bottom (**A**) or front (**B**) side, in which N52 helices (blue) form an interior, trimeric coiled coil with three hydrophobic grooves, three IPB19 helices (orange) pack into each of the grooves in an antiparallel orientation. (**C-F**) Detailed interactions between IPB19 and N52. The residues critical for the interactions are shown in sticks and labelled. The solid lines represent salt bridges, the dashed lines represent hydrogen bonds. (**G**) Sequence illustration of IPB19 binding. A single IPB19 peptide interacting with two N52 helices is shown in a sequence map. The blue solid lines represent salt bridges, the black dashed lines represent hydrogen bonds, and residues marked in red represent hydrophobic interactions. The residues in the C-terminal of IPB19 and the N-terminal of N52 that could not be visualized due to too low electronic density are underlined.

To understand the mechanism of the IPB19-based new inhibitors, we sought to determine the crystal structure of IPB19 complexed with N52. Two peptides were equally dissolved in denaturing buffer, and the mixture was dialyzed to allow refolding of the peptides. The N52/IPB19 complex was purified by size-exclusion chromatography and then crystallized in a hanging drop vapour diffusion system at 23 degree. The crystals belonged to the space group of H32, contained one pair of the N52/IPB19 peptides per asymmetric unit, and diffracted X-ray to a resolution limit of 1.24Å. We could build most of the residues of the peptides in the electron density map, but it was disappointed that the C-terminal MPER amino acids of IPB19 could not be located in the electron density map, reflecting flexibility of this region. Nonetheless, the overall core structure as a classical 6-HB conformation still revealed the critical interactions between IPB19 and N52. Owing to high resolution, we were able to observe extensive nonpolar and polar interactions between those peptides. As shown in Figure 8(A,B), three N52 helices formed an interior, trimeric coiled coil with three hydrophobic grooves and three IPB19 helices packed into each of the grooves in an antiparallel orientation, in which a cluster of hydrophobic residues (V1176, V1177, I1179, I1183, L1186, V1189, A1190, L1193, and I1198) of IPB19 make hydrophobic contacts with the N52 surface. In addition, we identified twelve hydrogen bonds stabilizing the 6-HB. Specifically, the N-atom of Ser-1175 donated a hydrogen bond to the OD1 of Asn-955, the ND2 of Asn-955 accepted a hydrogen from O-atom of Ser-1175, and these two residues were connected by hydrogen bonding via an ordered water molecule. Asn-1178 formed a hydrogen bond though a water molecule to Lys-947; Glu-1182 accepted a hydrogen bond from the OG-atom of Ser-943 (Figure 8(C)). Both Asn-1192 and Ser-1196 accepted hydrogen bonds from Lys-933, and both Glu-1195 and Leu-1197 accepted hydrogen bonds from Gln-926 (Figure 8(D)). Val-1177 donated a hydrogen bond to OD1-atom of Asn-953, while the ND2 of Asn-953 donated a hydrogen bond to the O-atom of Val-1177; Ile-1179 donated a hydrogen bond to OE1-atom of Gln-949, while the NE2-atom of Gln-949 donated a hydrogen bond to the OE1 of Gln-1180; Asn-1187 and Ser-939 were connected by hydrogen bonds involving a water molecule (Figure 8(E)). Both Ala-1190 and Asn-1194 accepted hydrogen bonds from the NE2-atom of Gln-935; Ile-1198 accepted a hydrogen bond from the ND2-atom of Asn928 (Figure 8(F)). Salt bridges can be formed between amino acids with positive and negative charges, and three pairs of salt bridges between N52 and IPB19 were observed between Glu-1195/Lys-933, Arg-1185/Asp-936, and Glu-1182/Lys-947 (Figure 8(G)). Taken together, the CD data and crystal structure revealed the mechanisms underlying the high binding and inhibition of the inhibitors.

## Discussion

In the urgency of the COVID-19 pandemic, considerable efforts have been focused on large-scale screening of existing drugs for repurposing [39–42]. Several approved antiviral therapies are the focus of clinical investigations, including lopinavir/ritonavir, danoprevir, remdesivir, and favipiravir [43–47]. Unfortunately, the clinical benefits of the selected drugs are very limited, which possibly attributed to their weak inhibitory activity (macomolar levels), thus calling for the *de novo* discovery of specific SARS-CoV-2 inhibitors that target the different steps of the viral life cycle. Toward this goal, we took immediate actions with multiple research projects and achieved significant findings [31,32,48–51]. We found that the S protein of SARS-CoV-2 has a much higher fusogenic activity than the S protein of SARS-CoV and its HR2-derived fusion inhibitors exhibits potent and broad-spectrum inhibitory activity against divergent human CoVs [31,32]. Interestingly, several pan-CoV fusion inhibitors, including EK1V1, EK1C4, and IPB02, were serendipitously found to have cross-inhibitory activities against divergent HIV-1, HIV-2, and simian immunodeficiency virus (SIV) isolates [51]. In this study, we continued our efforts to develop SARS-CoV-2 fusion inhibitors with improved pharmaceutical profiles. We first demonstrated that the spike fusion protein membrane-proximal external region (MPER) plays important roles in SARS-coV-2 cell fusion and infectivity and that IPB19 peptide comprising of the MPER sequence is an ideal template for design of the membrane fusion inhibitors. Accordingly, a group of novel fusion inhibitor lipopeptides was rationally designed and characterized. It was found that cholesterol-conjugated lipopeptides with a polyethylene glycol linker possesses highly potent activity in inhibiting SARS-CoV-2 and its emerging variants, including those with the major mutations in circulating B.1.1.7 and B.1.351 strains. Similar to the previously reported IPB02-based lipopeptides [32], the newly developed inhibitors also exhibited the cross-inhibition on divergent human CoVs, including SARS-CoV, MERS-CoV, HCoV-NL63, and HCoV-229E that were tested in this work. Moreover, the strcutual properties of the new inhibitors were characterized by CD spectroscopy and crystallographic method, verifying the high-affinity binding of the inhibitors with the target mimic HR1-derived peptide N52. Although the crystal structure of IPB19 in complex with N52 could not visualize the binding of the MPER sequence due to the low electronic density, the structure did reveal the core interactions of the peptide with the targeting site in details. Combined, the data presented here are very informative to the structure and function of the S2 fusion protein and offer a new strategy for the development of broad-spectrum anti-CoV agents.

Depending on the cell type and CoV strain, S protein-mediated viral fusion/entry can occur either at the cell surface membrane or in the endosomal membrane and requires priming by cellular proteases. In the cases of SARS-CoV and SARS-CoV-2, while the S2′ cleavage via the transmembrane protease serine 2 (TMPRSS2) leads to fusion with the plasma membrane, the virus particles that are endocytosed following ACE2 binding can use endosome-residing proteases for fusion with the endosomal membrane. Previous studies suggest that SARS-CoV enters the target cells mainly via the endosome fusion pathway, where its S protein is cleaved by endosomal cysteine proteases cathepsin B and L, but it also employs TMPRSS2 for S protein priming epecially in primary target cells [52–55]. Differently, SARS-CoV-2 harbours a S1/S2 cleavage site in the S protein and induces typical syncytium formation in infected cells [34,56], suggesting that the plasma fusion pathway might dominate its cell entry. Indeed, the cell entry of SARS-CoV-2 requires TMPRSS2 for S protein priming and can be specifically blocked by TMPRSS2 inhibitors [9]. A furin-mediated S1/S2 precleavage in infected cells may promote subsequent TMPRSS2-dependent cell entry, similar to a mode adopted by MERS-CoV [57,58]. Whatever the pathway is, lipopeptides are particularly attractive for the development of fusion inhibitors against CoVs because they can enhance inhibition of viral fusion both at the plasma membrane and within endosomal compartments. In this regard, we previously demonstrated that HIV fusion-inhibitory lipopeptides can bind to both viral and cellular membranes [24,27]. It is conceivable that while the cell surface binding enhances efficacy against viruses via the plasma fusion pathway, the lipopeptide bound to the virions are also taken up via the endocytosis thereby enabling activity against viruses that do not fuse until they have been endocytosed. The lipopeptide strategy was also applied to develop effective fusion inhibitors for influenza virus [23,59], which employs the endosome fusion pathway for cell entry.

Our previous studies demonstrated that addition of a space linker between the peptide sequence and lipid moiety can either improve or impair the antiviral potency of different fusion inhibitors [22,26,28,29], highlighting a successful strategy should position the peptide to overcome the steric hindrance in a right place. Different from the IPB02 derivatives that require a relatively longer PEG8 linker, we found that IPB19-based lipopeptides require a short PEG4 linker, reflecting the structure–activity relationship (SAR) of the HR2-derived fusion inhibitor lipopeptides. In order to develop broad-spectrum CoV fusion inhibitors, Xia *et al*. initially characterized the peptides derived from the HCoV-OC43 HR2 domain, resulting in a pan-CoV inhibitor EK1 with sequence substitutions [16]. In the outbreak of the COVID-19, a panel of EK1-based lipopeptides was recently created with improvements, and of them the most potent inhibitor EK1C4 was introduced with two space linkers (GSGSG and PEG4) between the EK1 peptide and a cysteine-linked cholesterol molecule [34]. Very recently, Outlaw *et al*. reported a SARS-CoV-2 HR2-derived lipopeptide containing the sequence similar to IPB02 and the GSGSG-PEG4 linker, which was ∼30-fold more potent than an EK1C4-like lipopeptide and potently blocked infection by live SARS-CoV-2 in human airway epithelial (HAE) cultures, an *ex vivo* model designed to mimic respiratory viral propagation in humans [33]. Importantly, the daily intranasal administration of a dimeric HR2 lipopeptide to ferrets completely prevented SARS-CoV-2 direct-contact transmission during 24-hour co-housing with infected animals, under stringent conditions that resulted in infection of 100% of untreated animals, thus suggesting a safe and effective intranasal prophylactic approach to reduce transmission of SARS-CoV-2 [60].

In our future studies, we will dedicate to address four related questions. First, while the HR1 sequences of SARS-CoV-2 and SARS-CoV display nine amino acid substitutions, the HR1 core sequences of other human CoVs possess more marked divergences, thus it is interesting to exploit the mechanism underlying the broad-spectrum anti-CoV activity of the SARS-CoV-2 derived fusion inhibitor peptides. In this regard, the HR1 domains might be conformationally conserved during the membrane fusion process. Second, while the IPB19-based lipopeptides can maintain their high potency in inhibiting the current circulating SARS-CoV-2 variants with the mutations in S1/RBD, the effects of naturally occurring mutations in the S2 subunit, especially in the inhibitor-binding HR1 site, should be investigated. Related to this point, the genetic barrier and underlying mechanism of the new inhibitors themselves in inducing drug resistance are also fundamentally important. Third, given the limitation of the crystal structure of the IPB19/N52 complex presented here, an intact structure that can elucidate the MPER-mediated interactions is highly appreciated. Last but the most important, the *in vivo* safety and therapeutic efficacy of the new inhibitors should be carefully evaluated in suitable animal models before advancing them to a clinical setting.

## Supplementary Material

Supplemental Material

## Data Availability

All data are fully available without restriction. The atomic coordinates and structure factorshave been deposited in the Protein Data Bank under the accession code:7EK6.
