# Supplementary material for: Structure-based design and characterization of novel fusion-inhibitory lipopeptides against SARS-CoV-2 and emerging variants
Source: Emerg Microbes Infect. 2021 Jun 18;10(1):1227–40. doi: 10.1080/22221751.2021.1937329 (PMC8216258; doi:10.1080/22221751.2021.1937329)
Supplement: Supplemental Material [file TEMI_A_1937329_SM8443.docx]

**Table S1. Data collection and refinement statistics**

| **Parameter** | **IPB19/N52** |
| --- | --- |
| **Data collection** |  |
| Space group | H32 |
| Cell dimensions |  |
| *a,b,c (Å)* | 38.23, 38.23, 317.33 |
| *α,β,γ (°)* | 90.00, 90.00, 120.00 |
| X-ray source | SSRF BEAMLINE BL19U1 |
| Wavelength | 0.98 Å |
| Data range | 32.93-1.24 (1.24-1.32) |
| Reflections unique | 25377 |
| *R_merge_* (%) | 5.8 (121.5) |
| *I/ σI* | 37.31 (2.10) |
| Completeness | 97.1 (83.5) |
| Redundancy | 31.47 (14.14) |
| **Refinement** |  |
| Resolution range | 32.93-1.24 |
| No.Reflections | 25347 |
| *R_work_^a^ /R_free_^b^* | 0.1731 / 0.1854 |
| Nonhydrogen atoms | 753 |
| Protein | 653 |
| Water | 100 |
| B-Factor averages | 26.50 Å^2^ |
| Root mean square deviation |  |
| *Bond length* | 0.004 Å |
| *Bond angles* | 0.645° |
| **Validation** |  |
| MolProbity score | 1.28, rating 91th percentile among structures of comparable resolution |
| % Favored regions and Outliers in Ramachandran plot | 100.0, 0.0, 0.0 |

^a^ R_work_ indicates ∑hkl‖F obs (hkl)∣-∣Fcalc(hkl)‖/∑hkl∣Fobs(hkl)∣.

^b^ R_free_ indicates the cross-validation R factor for 5% of reflections against which the model was not refined.
